# Research on efficient feature extraction: Improving YOLOv5 backbone for facial expression detection in live streaming scenes

**DOI:** 10.3389/fncom.2022.980063

**Published:** 2022-08-10

**Authors:** Zongwei Li, Jia Song, Kai Qiao, Chenghai Li, Yanhui Zhang, Zhenyu Li

**Affiliations:** ^1^School of Economics and Management, Shanghai Institute of Technology, Shanghai, China; ^2^School of Management Science and Engineering, Anhui University of Technology, Maanshan, China; ^3^Business School, East China University of Science and Technology, Shanghai, China

**Keywords:** model optimization, object detection, attention mechanism, cascade classifier, live streaming

## Abstract

Facial expressions, whether simple or complex, convey pheromones that can affect others. Plentiful sensory input delivered by marketing anchors' facial expressions to audiences can stimulate consumers' identification and influence decision-making, especially in live streaming media marketing. This paper proposes an efficient feature extraction network based on the YOLOv5 model for detecting anchors' facial expressions. First, a two-step cascade classifier and recycler is established to filter invalid video frames to generate a facial expression dataset of anchors. Second, GhostNet and coordinate attention are fused in YOLOv5 to eliminate latency and improve accuracy. YOLOv5 modified with the proposed efficient feature extraction structure outperforms the original YOLOv5 on our self-built dataset in both speed and accuracy.

## Introduction

A new generation of marketing based on live streaming media through visual and auditory impacts has increased the appeal of shopping to all members of society. Compared with traditional marketing approaches, live marketing conveys richer sensory cues to consumers through real-time interactions, influencing their perceptions and willingness to purchase products. This live marketing can be considered as an investment in consumers' experience through the sensory cues of digital media (Chen et al., 2021).

The visual experience delivered to consumers by live demonstrations plays a vital role in consumers' attention. The facial expressions of anchors selling products are frequently a crucial area where audiences allocate their visual attention, directly affecting their emotions and perceptions (Simmonds et al., 2020). Exploring the important role that facial expression cues play in consumer perception, judgment, and purchase intention provides a theoretical contribution to the emerging field of sensory marketing. Most cutting-edge facial expression recognition and detection algorithms are limited to available standard facial expression datasets in the laboratory, but facial expression detection is more complicated because of various backgrounds and lighting in actual live streaming scenarios. When deploying these deep learning models on embedded/mobile terminals, real-time detection is difficult on the limited available CPU and GPU resources. Therefore, a strictly accurate and quick detection model is fundamental to analyzing sensory marketing and encounters significant challenges.

Recognizing other people's facial expressions and understanding their emotional implications is an advanced human ability that processes the rich information captured by their visual system. The increasing use of machine vision and neural networks makes it possible for machines to acquire the same capability to help achieve self-cognition. In Li et al. (2020), the automatic facial expression detection method combining local binary pattern (LBP) features and the attention mechanism had high detection accuracy. However, the experimental data are all derived from standard facial expressions in a laboratory environment, which is hard to simulate facial expression changes in reality. Mollahosseini et al. (2016) first applied the inception layer architecture to the network and successfully realized facial expression detection across datasets to generalize the model. Because of insufficient feature extraction, it cannot compete with other complex convolutional neural networks (CNNs). Practicality becomes the primary factor for model development, considering the continuous increases in the demands of facial expression detection. Sudha et al. (2015) released a facial expression detection system for installation on a mobile phone. However, because of the high computational complexity and insufficient GPU capability, the task of real-time detection is difficult. Pei and Shan (2019) utilized a deep convolutional network (DNN) to probe the facial micro-expressions of students during a class period. By decomposing the frames of the actual course video, detecting the facial markers of the students, and extracting the optical flow features, the monitoring of students' attention in class was realized. Because of the excessive computational consumption required by optical flow feature extraction, the detection delay was apparent, and cannot meet the needs of real-time detection.

In summary, although many investigators have performed significant research on facial expression detection, there are still problems such as deficient datasets, limited computing resources, and insufficient model feature extraction in specific applications. This paper proposes an improved object detection model based on the above research. First, we provide a variety of samples for model training after data preprocessing. Then, we choose the typical one-stage detector YOLOv5 as the benchmark network and use the Ghost module (Han et al., 2020) to replace the backbone feature extraction. Additionally, we add coordinate attenuation (CA) (Hou et al., 2021) for backbone feature strengthening, which focuses the limited computational resources on the object regions. The experiments show that the proposed model can achieve optimal precision while reducing the model to approximately half its original size.

The key contributions of this work are as follows:

- A dataset of anchor facial expressions is established, filling the data gap for live-streaming facial expression detection.
- A two-step cascade classifier and recycler is designed for filtering images to effectively remove invalid samples with missing and incomplete faces in live videos.
- A lightweight and high-precision anchor facial expression detection model is presented. We integrate the Ghost module and CA into YOLOv5 to realize detection accuracy and speed improvements.

The remainder of this paper is structured as follows. In Section Related work, we provide an overview of the evolution of the YOLO network and the development of the attention mechanism. Section Data preprocessing develops a data preprocessing methodology to collect facial expression data from Chinese live streaming marketing videos, and Section The improved YOLOv5 algorithm presents the improved YOLOv5 model. A set of comparison experiments and analyses between our model and others for objective evaluation are provided in Section Experiments. Finally, Section Conclusions and future work concludes the work and explores future research priorities.

## Related work

Lightweight but efficient feature extraction architecture contributes toward better and faster progress in YOLO. In the following subsections, we revisit the basics of the YOLO network. In particular, we analyze the corresponding techniques concerning deep learning. We introduce how the attention mechanism provides an alternative for enhancing model performance.

### Feature extraction in YOLO

Overall, the object detection algorithm for facial expression consists of two main procedures: feature extraction and feature classification. Because the classification effectiveness depends on the features produced by the extraction procedure, it is vital to design an efficient feature extraction structure. Before deep learning was involved in this task, traditional methods such as the Gabor wavelet (Kyperountas et al., 2010), LBP (Ojala et al., 1996), and optical flow (Yacoob and Davis, 1996) methods were used to extract the appearance features in images. However, these methods possess significant limitations, such as excessive computation and constrained feature definition.

**Figure 1:**
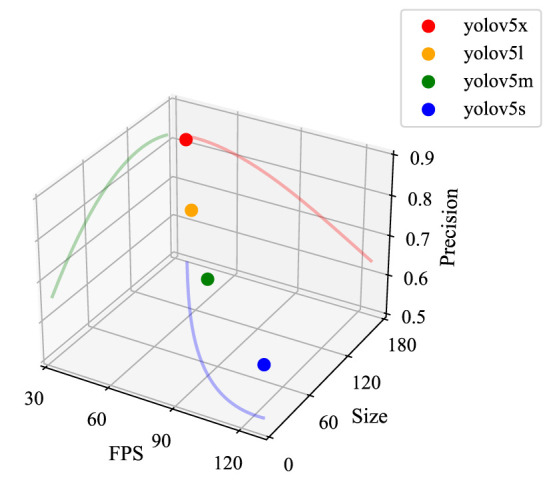
Performance of different YOLOv5 networks. The experimental dataset is COCO128, and the experimental environment is the Jetson Nano edge computing device. FPS is the number of images that can be detected per second; Precision is the detection accuracy (mAP@0.5); the Size units are MB.

Over the years, convolutional neural networks (CNNs) have nearly completely replaced traditional feature extraction methods as the mainstream framework and machine vision methods because of their outstanding feature expression capabilities. Thus, the performance of the best object detector has improved steadily over time. It is well-known that simply stretching the width and depth of the network does not improve the network performance directly and effectively. On the contrary, this approach resulted in a series of problems involving high computational complexity, overfitting tendency, and gradient divergence. The Inception module of GoogLeNet (Szegedy et al., 2015) combined different convolution layer sizes and pooling operations to improve the size-adaptability of the network. They also added a 1 × 1 convolutional kernel to decrease the dimensionality of feature layers, significantly reducing the model's complexity. The innovative network design of GoogLeNet laid the foundation for the research on lightweight convolutional neural networks. YOLOv1, proposed by Redmon et al. (2016), was based on the network structure of GoogLeNet but replaced the Inception module with 1 × 1 reduced layers and 3 × 3 convolutional layers. It is a lightweight design framework that facilitates a high-speed image inference speed. However, the location accuracy in YOLOv1 was lower than for another classical object detection algorithm, R-CNN (Girshick et al., 2014). Redmon and Farhadi (2017) designed Darknet-19 in YOLOv2 by combining the advantages of networks such as VGG16 (Simonyan and Zisserman, 2014). YOLOv2 has better performance than YOLOv1, even though the network was lighter. With the introduction of ResNet (He et al., 2016), YOLOv3 incorporated the residual structure and expanded the former network into Darknet-53, which consisted of many 1 × 1 and 3 × 3 convolutional layers (53 layers in total) stacked consecutively. The residual structure can alleviate the problems of gradient explosion and gradient dispersion caused by the deepening of the network, while the feature pyramid network (FPN) (Lin et al., 2017) was introduced to enhance feature fusion. Because there was still a gap between YOLOv3 and the faster R-CNN, YOLOv4 (Bochkovskiy et al., 2020) was developed to provide further enhancements. Based on extensive experiments, diverse detection techniques were tried using YOLOv4 to provide a possible solution to the mismatch between inspection accuracy and speed. However, with the continuous advance of algorithms, YOLOv5 completely superseded YOLOv4 because of its ultra-fast real-time object detection speed. Initially, YOLOv5 provided four different network structures (YOLOv5x, YOLOv5l, YOLOv5m, and YOLOv5s). By controlling the width and depth of the extracted features, the network can meet different object detection arrangement needs. As shown in Figure 1, YOLOv5x has the highest detection accuracy, which is attributed to its wider and deeper feature maps under the same experimental conditions, even though it has more parameters, higher model complexity, and longer detection times. Conversely, YOLOv5s has the lightest network and the fastest detection speed but the lowest detection accuracy, making it suitable for real-time detection applications with higher detection speed requirements.

### Attention mechanism in object detection

The concept of attention mechanism was first pointed out in the academic literature (Mnih et al., 2014) as vision attention in a neural network model to adaptively process image regions at high resolution.

Subsequently, the attention mechanism demonstrated its advanced interpretability in natural language processing, renewing intense interest by researchers and significantly impacting machine vision tasks. The attention mechanism can be conveniently embedded in deep learning networks as a structure that can reinforce feature information to increase detection accuracy. Fundamentally, it is a process of allocating higher weights to the object regions of interest to carry out a dynamic transfer of limited computational resources. The lightweight Squeeze-and-Excitation (SE) attention (Iandola et al., 2016) allowed the network to assign different weights to each channel, emphasizing the important features containing rich information and diminishing unimportant features through squeezing and expanding operations. Convolutional block attention module (CBAM) (Woo et al., 2018) is a bi-directional concentration method that performs global average pooling in the spatial dimension and global maximum pooling in the channel dimension. Nevertheless, good multi-object detection makes it equally necessary for the attention mechanism to calculate the ratio of global average pooling to global maximum pooling. Miao et al. (2022) established a novel cross-contextual attention-guided network (CCAGNet). They introduced 3 different attention mechanisms to guide the network for learning area focusing by simultaneously considering contextual information about multiple areas, including adjacent, intersection, spatial, and channel areas. While the extra burden of this operation is minor for a large network, the success cannot be copied in a lightweight network.

CA was made-to-measure for mobile networks, as presented by Hou et al. (2021). Unlike CBAM, which forces channel compression, CA adaptively reduces the channel dimension in the structure's bottleneck at a reasonable rate to avoid the loss of important information. At the same time, CA can furnish more comprehensive spatial information through two complementary one-dimensional global pooling blocks, which is more favorable for optimizing feature extraction structures.

## Data pre-processing

Most images in static facial expression databases are by researchers deliberately making standard facial expressions in their laboratory settings. However, such images are not conducive to the dynamic understanding of different degrees of facial expressions in videos, such as FER-2013 (Giannopoulos et al., 2018) and AFEW (Yu and Zhang, 2015). By contrast, it has been shown that datasets composed of video sequences such as CK+ and MMI contain the dynamic multiple facial expression changes that are more suitable for dynamic recognition and detection of facial expressions in videos (Pantic et al., 2005; Lucey et al., 2010). This paper selects multiple live videos of four anchors as the data source for a self-built dataset to bridge the gap of facial expression data in live streaming media scenes. However, it is challenging to construct complex data present to the classifier even for the same anchor while avoiding over-fitting because of varying scenes, makeup styles, and lighting.

Because there are many invalid frames with missing and partially obscured faces in a video, we established a cascade classifier to objectively and effectively filter the picture frames. The filtered images constitute a live streaming facial expression database, and facial expression classification and location annotation are performed on these images.

### Two-step cascade classifier and recycler

Not all frames are equally important in a complete live video. There are situations in which the anchors leave the live room, show product details using zoomed-in views, and turn to interact with participants during a live broadcast. Therefore, the corresponding video frames fail to provide sufficient feature information for facial expression training and detection, indicating that it is necessary to distinguish missing and obscured faces and profiles that may occur at any time in the video.

The cascade classifier based on the AdaBoost algorithm (Viola and Jones, 2001) is one of the most commonly used facial detection algorithms and has a reputation for high-speed detection. The process of establishing a classical cascade classifier consists primarily of two parts: the training of weak classifiers and the cascading of strong classifiers (Luo, 2005; Oliveira et al., 2005). The weak classifiers are trained iteratively to obtain the optimal weak classifiers with appropriate thresholds, and then the AdaBoost algorithm combines these optimal weak classifiers to generate the strong classifier.

The strong classifier generation formula can be expressed as


(1) $$\begin{array}{l} h\left(\alpha \right)=\{\begin{array}{cc} 1 & \sum_{t=1}^{T}\theta_{t}h_{t}\left(\alpha \right)\geq \;\frac{1}{2}\;\sum_{t=1}^{T}\theta_{t}\\ 0 & otherwise \end{array} \end{array}$$

where θ_*t*_ is the error rate of the weak classifier, *h*_*t*_ is the feature classifier with the lowest error rate, and *T* represents the number of optimal weak classifiers. Then, we combine the strong classifiers with high detection rates into the final filtered cascade classifier through cascading operations.

When performing object detection, the cascade classifier applies Haar-like features (Lienhart and Maydt, 2002) to quantify facial features as characteristic vectors and computes multi-scale and multi-region feature values for the input image. Because this switching process requires tremendous computation, we adopt the integral image to quickly find the pixel sum of all regions in the image. The computational process of the integral image can be defined as


(2) $$\begin{array}{l} S\left(\alpha ,\beta \right)=\Sigma_{\alpha^{\prime }<\alpha ,\beta^{\prime }<\beta }I\left(\alpha ,\beta \right) \end{array}$$

where *I*(α, β) denotes the pixel value at (α, β) and *S*(α, β) is the sum of all pixels in the direction of the upper left corner of the original image (α, β).

Compared with the feature values in the strong classifier, the next round of judgment to achieves the effect of filtering classification only when the threshold of calculated values is satisfied. However, because the threshold division of the strong classifier affects both the high pinpoint rate and misjudgment probability, the recognition accuracy of cascade classifiers remains coarse.

**Figure 2:**
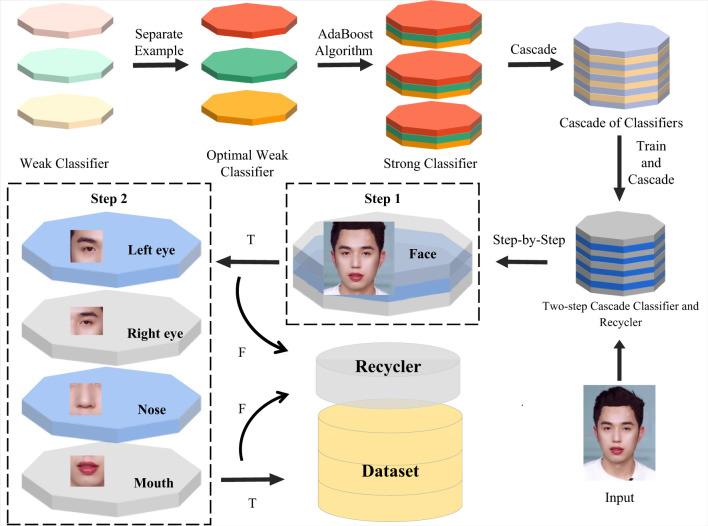
Data preprocessing algorithm. First, the images of face region and five senses are trained separately to obtain each cascade classifier. Then these cascade classifiers are combined to form a two-step cascade classifier and recycler. Finally, the two-step cascade classifier and recycler rejects the invalid images in two stages.

To ensure that each clip in the final database retains complete facial information, we establish a two-step cascade classifier and recycler for detecting facial contours and details in stages to remove invalid frames in videos quickly and accurately. First, we used positive and negative face sample training data and five characteristic features to obtain cascade classifiers for recognizing faces, left eyes, right eyes, noses, and mouths, respectively. Then, these cascade classifiers were further cascaded to form a two-step cascade classifier and recycler with double insurance. In the first stage, the cascading classifier removes the images without a face region and otherwise retains the filtered images for pending processing. In the second stage, the cascade classifier group recognizing the five senses is utilized to further filter the images retained in the first stage. In this regard, these cascade classifiers can be abstracted as the judgment nodes of the decision tree, where only images with all the above characteristic features are judged as acceptable to keep while the others are not. The resulting two-step cascade classifier and recycler is formulated as shown in Figure 2.

Relying on the two-step cascade classifier and recycler, a processed facial expression database of live streaming media scenes emerges quickly, providing helpful feature samples for model training and inference. The methodological approach proposed appears to be advantageous for improving the precision of the model.

### Facial expression classification

**Figure 3:**
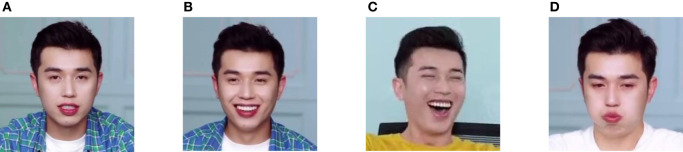
Sample diagrams of facial expression classifications: **(A)** Attentive; **(B)** Happy; **(C)** Excited; **(D)** Funny.

After filtering the dataset, the pictures must be classified and labeled manually. In this paper, the anchors' facial expressions are divided into four categories significant for exploring the emotional cues conveyed by anchors to consumers (refer to Figure 3 for examples). These four categories are Attentive, Happy, Excited, and Funny, described as follows:

- Attentive: the exhibition of facial expression when anchors interpret the details of the product professionally and intently.
- Happy: smiling facial expressions presented by anchors to win consumers' preferences.
- Excited: anchors' enthusiastic and laughing facial expressions that drive consumers' emotions and stimulate their desire to buy.
- Funny: deliberate negative facial expressions by the anchor, such as dislike, sadness, and anger, to create a sense of contrast and an entertaining and funny atmosphere. These expressions are relatively rare and contain the same intention, so we group them into a single category.

## The improved YOLOv5 algorithm

YOLOv5s, the lightest version of YOLO, is selected as the baseline network to be improved in this paper. Based on this, both improvements to weight and precision are made to achieve a balance between speed and accuracy.

The network architecture of the original YOLOv5s is composed of four main parts: the Input, Backbone, Neck, and Prediction layers. The images first pass through the Input layer, where some of the same methods from YOLOv4 remain (e.g., mosaic data enhancement and auto-learning bounding box anchors). Then, the Backbone uses focus downsampling, the improved cross stage partial (CSP) structure, and the spatial pyramid pooling (SPP) structure to extract the feature information of pictures. In the Neck, YOLOv5's “double tower tactic,” i.e., the path aggregation network (PAN) (Liu et al., 2018) and FPN, are used to strengthen feature fusion successfully. Finally, the Prediction layer draws up the prediction information of images (i.e., coordinate information of bounding boxes, prediction confidence, and classes of an object).

**Figure 4:**
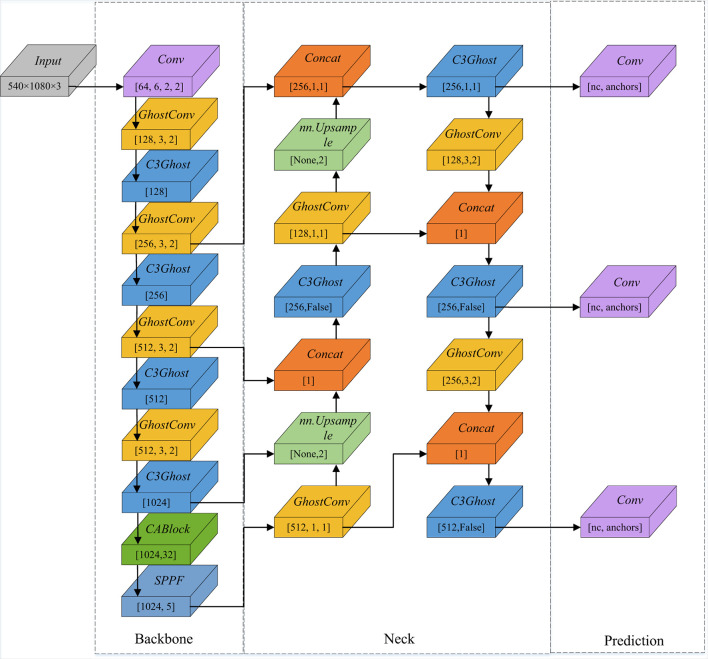
Improved YOLOv5 framework. C3Ghost replaces the C3 module, and Ghostconv replaces Conv in the original network. In addition, CABlock is placed before the SPPF module.

The original YOLO network still suffers from several limitations because of high computational requirements and inadequate feature extraction in the Backbone. Our improved network aims to optimize the mismatch between reduced weight and high accuracy. The GhostNet (Han et al., 2020), referring to C3Ghost and Ghostconv, is selected for incorporation into the Backbone, and CA (Hou et al., 2021) is chosen to enhance the attention of the network. The improved YOLOv5 framework is illustrated in Figure 4.

### Lightweight structure: GhostNet

**Figure 5:**
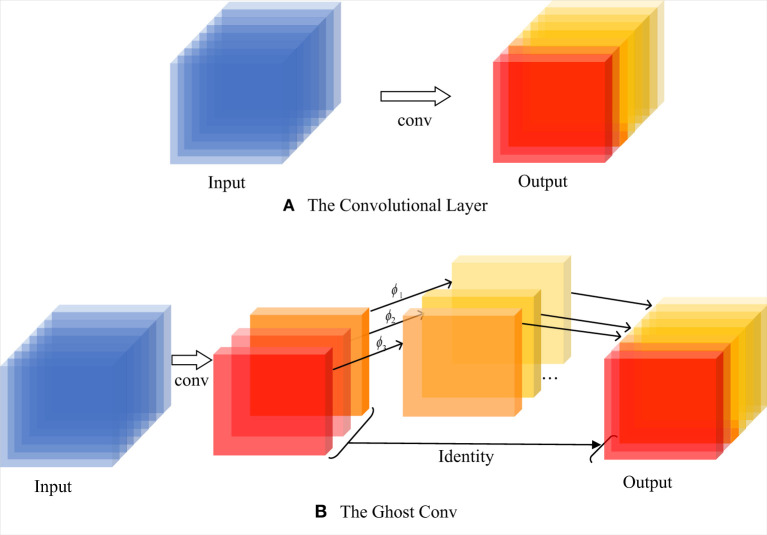
The process of traditional convolution and Ghost convolution: **(A)** the original convolution process; **(B)** the convolution structure specific to GhostNet.

To obtain a more lightweight implementation, we modify the original model by using a lightweight network model, GhostNet, which dramatically reduces the number of computational parameters by eliminating redundant feature maps. GhostNet primarily consists of a two-step process of integrating the original convolution: (i) generation of partial feature maps with fewer convolution kernels; (ii) a simple linear transformation of feature maps to obtain additional Ghost feature maps. These two sets of feature maps are stitched to output together. Figure 5 illustrates the transformation process of the feature maps.

**Figure 6:**
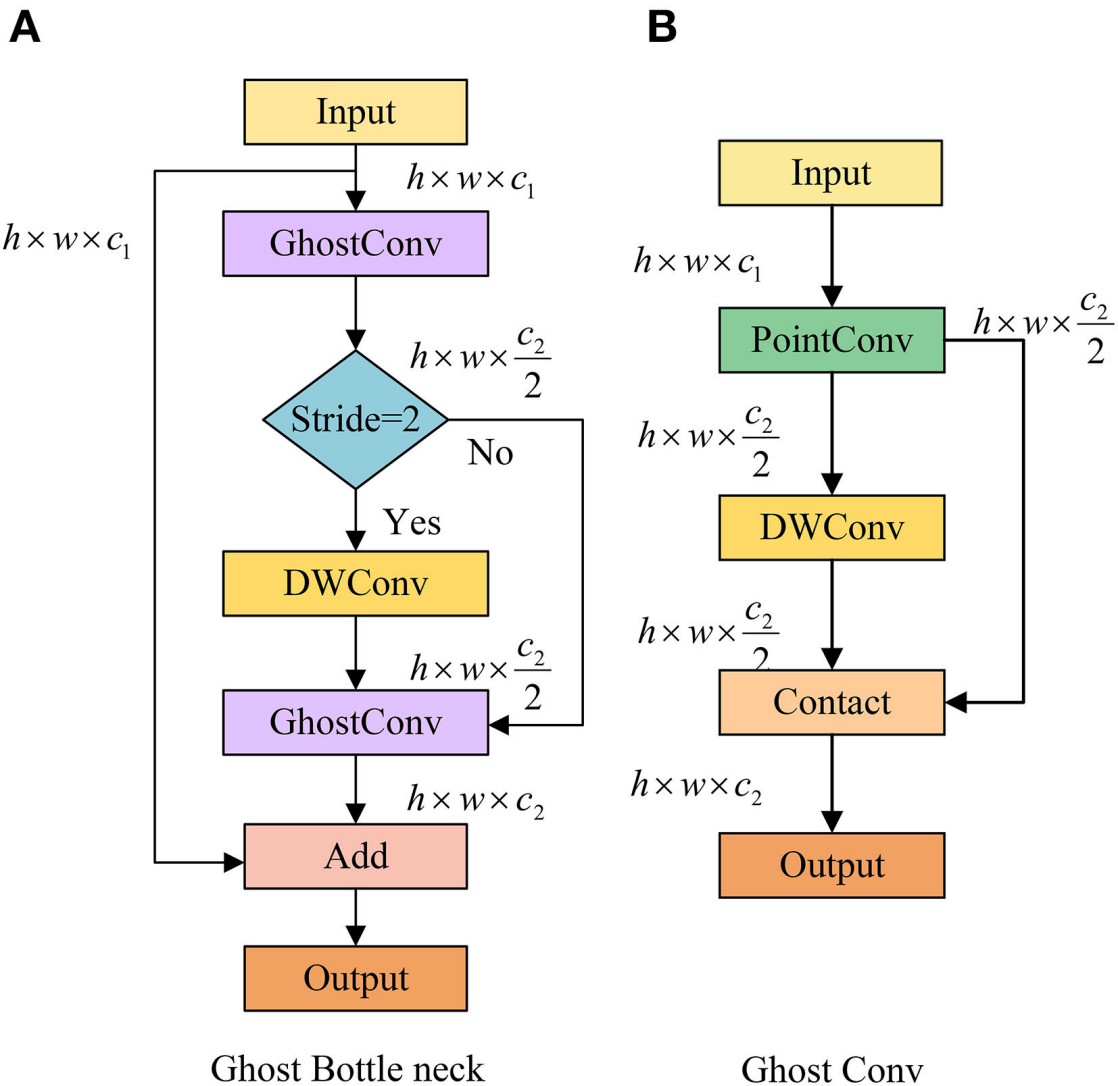
Network structure of **(A)** GhostBottleNeck and **(B)** Ghost convolution.

GhostBottleNeck is composed of two different GhostConv layers (see Figure 6A). The first GhostConv plays a vital role in the expansion of channels, whereas the second GhostConv is used for matching output by cutting channels. In addition, when the stride is 2, depthwise-separable convolution (DWConv) can convert the shape of the feature map. Within the GhostConv structure (see Figure 6B), the feature map first undergoes a 1 × 1 point convolution for cross-channel feature extraction, where the number of channels is reduced to half of the original in this case. Then, feature extraction across feature points is performed by a 5 × 5 DWConv, and the other half is obtained. The final output is a concatenation of the results generated by these two parts.

Based on GhostBottleNeck, we constitute a new C3Ghost module to replace the original C3 module in the YOLOv5 network and replace the Conv of the original YOLOv5 by GhostConv. These modifications guarantee a more lightweight implementation while reducing the convolutional layer parameters.

### Attention mechanism: Coordinate attention (CA)

Motivated by the goal of maintaining high accuracy with a smaller model size, we incorporate CA into the benchmark network of YOLOv5, which considers not only the relationship between channels but also the location information in feature space. Incorporating CA allows the neural network to obtain larger area information while avoiding a larger overhead introduction.

**Figure 7:**
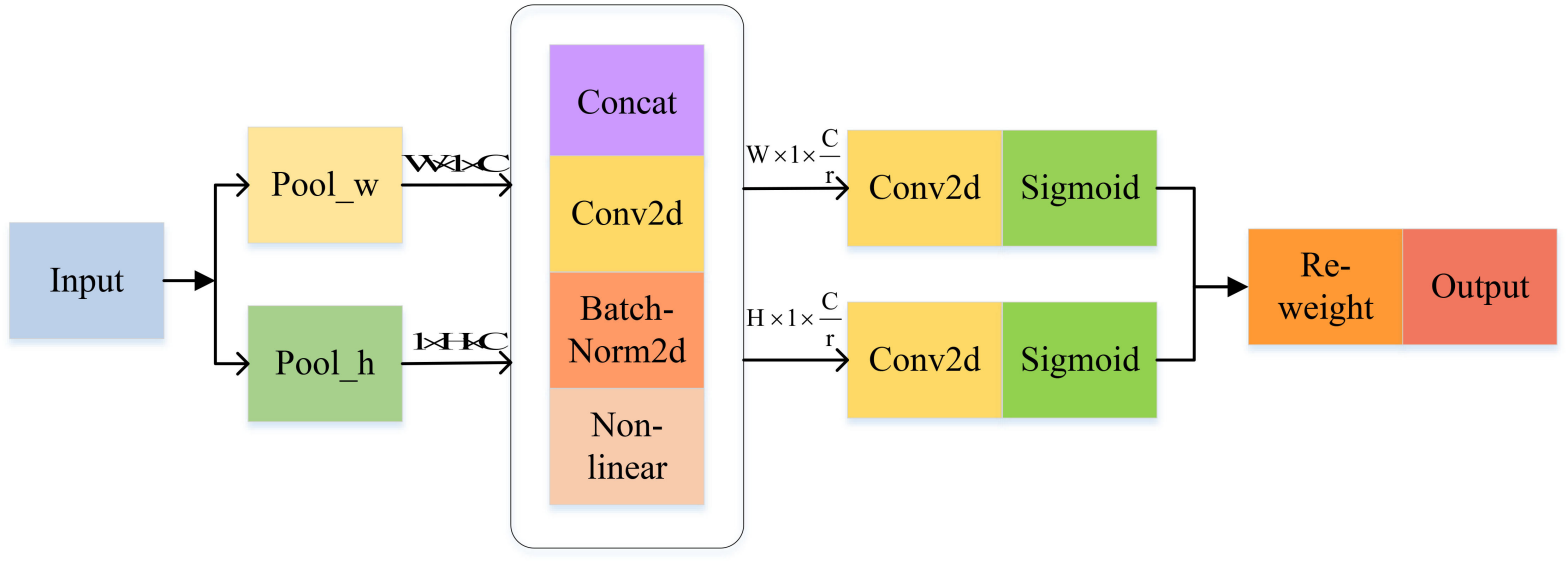
CA attention mechanism.

The main task of CA is to encode channel attention by aggregating features in two directions. This contributes to retaining location information along one direction and capturing long-term dependencies along the other direction, complementing feature information and enhancing the expression capability of objects of interest. CA can be divided into two consecutive processes: coordinate information embedding and coordinate attention generation (see Figure 7).

#### Coordinate information embedding

In general, when generating channel attention, the spatial information is usually decoded by two-dimensional global pooling, but it also comes with the absence of location information. Two parallel one-dimensional feature encodings are added to solve this problem, incorporating spatial coordinate information into the generated attention maps. Specifically, with a given feature tensor, CABlock uses two different pooling kernels of size (H, 1) and (1, W) to encode the feature descriptors in the horizontal and vertical directions, respectively, as shown in Equations (3) and (4):


(3) $$\begin{array}{l} z_{c}^{h}\left(h\right)=\frac{1}{w}\Sigma_{0≦\alpha ≦W}x_{c}\left(h,\alpha \right) \end{array}$$

(4) $$\begin{array}{l} z_{c}^{w}\left(w\right)=\frac{1}{H}\Sigma_{0≦\beta ≦h}x_{c}\left(\beta ,w\right) \end{array}$$

where *h* and *w* represent the height and width of feature maps, respectively, *x*_*c*_ is the input feature map of *x* in channel *c*, and $z_{c}^{h}\left(h\right)$ and $z_{c}^{w}\left(w\right)$ are the directional awareness of *x*_*c*_ in the horizontal and vertical directions, respectively. The above transformations result in a pair of complementary direction-aware feature maps, allowing CA to maintain long-term reliance on one spatial direction and preserve accurate location information in the other, leading to a higher concentration of attention on the located area.

#### Coordinate attention generation

After obtaining the position information in two directions, the features are concatenated, convolved, and activated sequentially to obtain the feature map *f*, generated by


(5) $$\begin{array}{l} f=RELU\left(conv_{1\times 1}\left(concat\left[z^{h},z^{w}\right]\right)\right) \end{array}$$

The feature tensors *f*^*h*^ and *f*^*w*^ are obtained after separating the features of *f* in the *H* and *W* directions and then making a 1 × 1 convolution on them to obtain the matchable attention weights *g*^*h*^ and *g*^*w*^, computed as


(6) $$\begin{array}{l} g^{h}=\sigma \left(conv_{1\times 1}\left(f^{h}\right)\right) \end{array}$$

(7) $$\begin{array}{l} g^{w}=\sigma \left(conv_{1\times 1}\left(f^{w}\right)\right) \end{array}$$

where σ is the activation function.

The final feature map *y* with weighted attention is obtained by individually weighting each value of the initial feature tensor *x*.


(8) $$\begin{array}{l} y_{c}\left(\alpha ,\beta \right)=x_{c}\left(\alpha ,\beta \right)\times g_{c}^{h}\left(\alpha \right)\times g_{c}^{w}\left(\beta \right) \end{array}$$

where *y*_*c*_ denotes the feature map of *x*_*c*_ after weighting.

CA is added to the backbone network of YOLOv5, maintaining the model detection at high accuracy with only a few computational cost, demonstrating the effectiveness of CA for network improvement.

## Experiments

### Dataset and experimental environment

In this paper, the proposed model is tested and trained with a self-constructed dataset. The facial expressions of anchors in this dataset are divided into four categories (attentive, happy, excited, and funny), totaling 2,395 images. The size of these images is 540 × 1,080. After data preprocessing, the database possesses more distinct facial features, favoring the improved model for a more advanced feature extraction process. Table 1 shows the classification and distribution of the dataset.

**Table 1:** Dataset category statistics.

	**Attentive**	**Happy**	**Excited**	**Funny**	**Total**
Train	423	426	373	309	1,531
Validation	77	76	66	57	276
Test	136	131	125	106	498

We deploy the improved model in a laboratory hardware system consisting of an NVIDIA GeForce RTX 3070 GPU, AMD Ryzen 7 5800X CPU, deep learning framework with PyTorch, and hardware acceleration with CUDA 12.0.

### Experimental results

#### Analysis of experimental results

For the accurate and objective validation of the improved model, we perform a series of comparative experiments on the self-constructed dataset. The experimental results are evaluated using the criteria of mAP, weights, GFLOPs, parameters, and accuracy density. mAP is a common measure of neural network accuracy with the model precision measured by mAP@0.5 and mAP@0.5:0.95 (Borisyuk et al., 2018). Weights, GFLOPs, and parameters measure models' size, complexity, and computational volume, respectively. Furthermore, in a recent benchmark test, a new indicator for performance measurement called the accuracy density was proposed (Bianco et al., 2018), defined as the accuracy divided by the number of parameters. The accuracy density can visually represent the balance between the parameters and accuracy of targeted models, so we adopt this criterion to evaluate the comprehensive performance of the model.

The test results are listed in Table 2. The mAP values of both models are maintained above 98%, proving that the data preprocessing preserves rich facial features in the images, enhancing the feature extraction ability of our models. Compared with the original model, the size and complexity of the improved model are reduced by about one-half, and the network parameters are reduced by 47.2%. Moreover, although mAP@0.5:0.95 declines slightly, the accuracy of the proposed model is significantly elevated, as reflected by being 0.4% higher in mAP@0.5 and 52.6% higher in accuracy density, demonstrating the improved model's validity.

**Table 2:** Comparison of experimental results of the original and proposed models.

**Model**	**mAP@0.5 (%)**	**mAP@0.5:0.95 (%)**	**Weights (MB)**	**GFLOPs**	**Parameters (M)**	**Accuracy density**
YOLOv5	98.4	84.9	15.4	15.8	7.020913	14.015271
YOLOv5_ Ghost_CA	98.8	84.5	8	8.1	3.708425	26.642038

**Figure 8:**
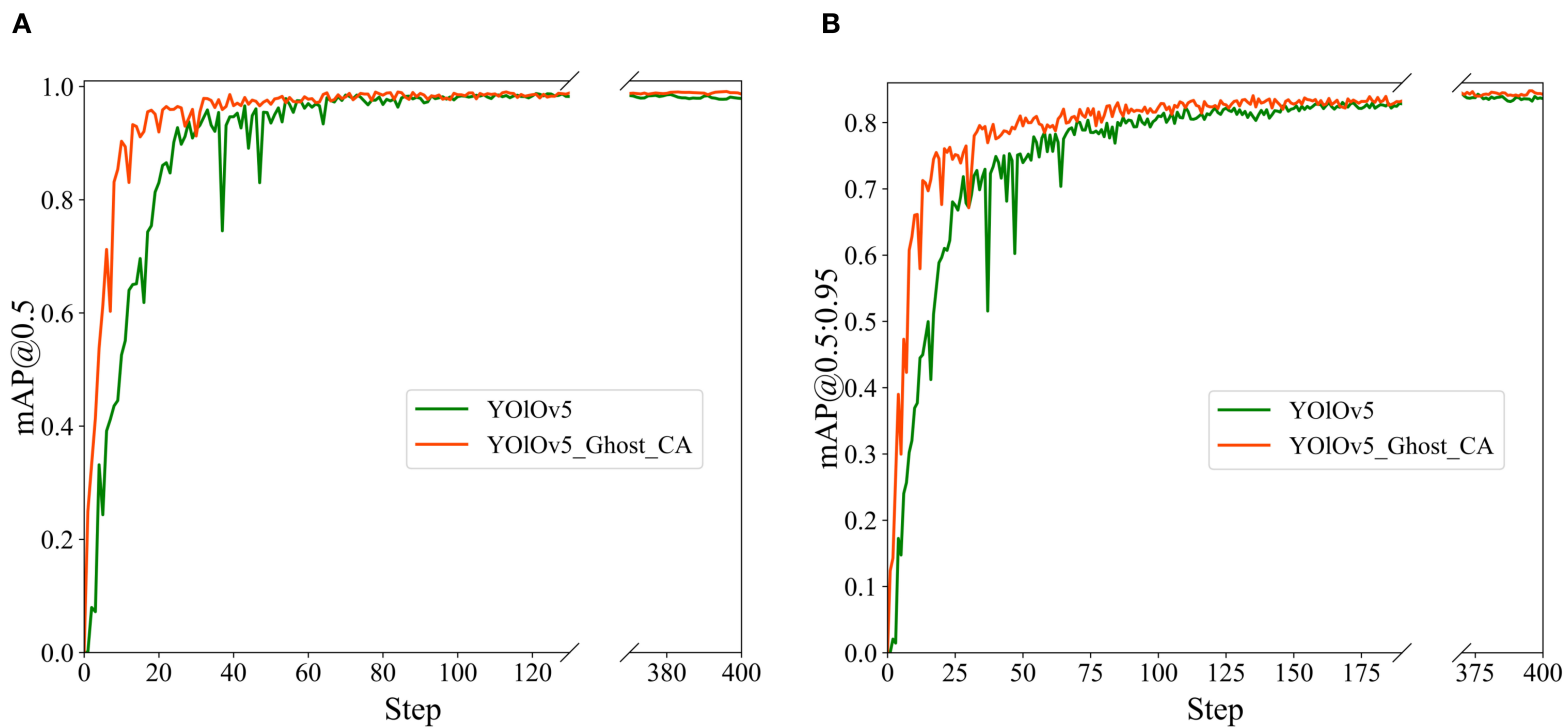
The mAP histories of the original and proposed YOLOv5 models: **(A)** mAP@0.5; **(B)** mAP@0.5:0.95.

In addition, we record the values of mAP@0.5 and mAP@0.5:0.95 for each iteration of the training process and illustrate the relevant graphs in Figure 8. The orange and green curves depict the accuracy of the proposed and original YOLOv5 models, respectively. Our model presents distinctly faster convergence.

**Figure 9:**
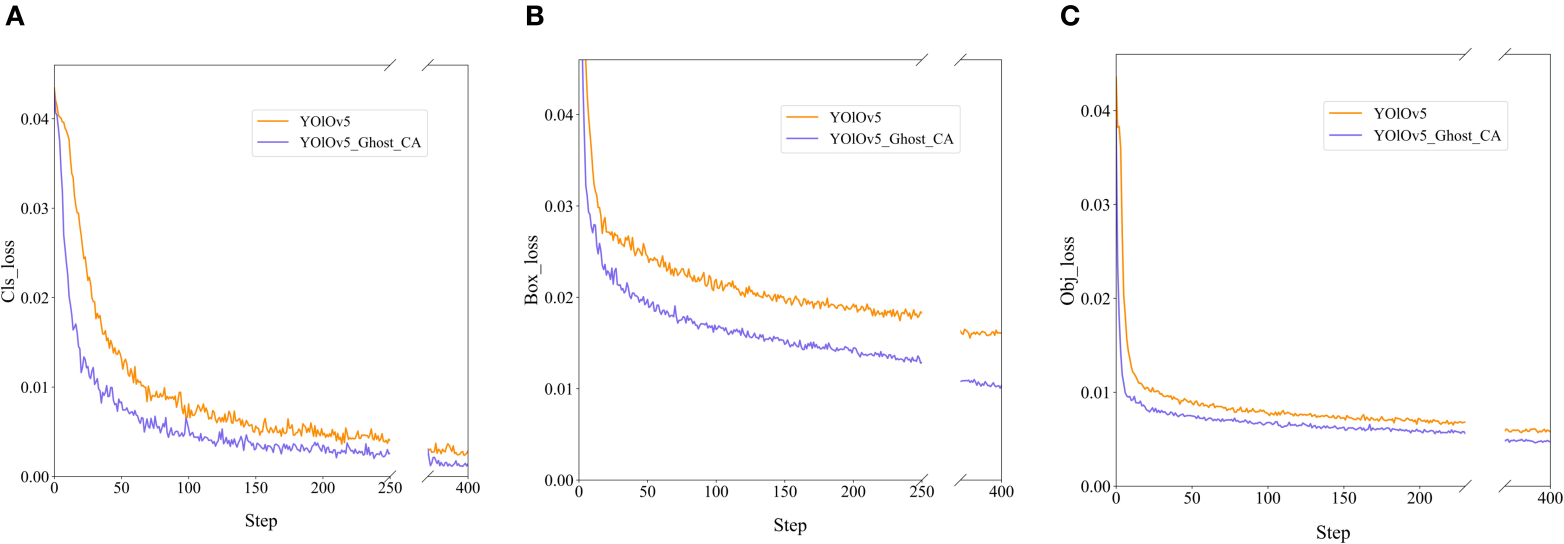
The losses of the original and improved YOLOv5 models: **(A)** *cls_loss*; **(B)** *box_loss*; **(C)** *obj_loss*.

We also record the loss values of the training model to calculate the difference between the predicted and true model values, including *cls_loss* for supervising category classification, *box_loss* for measuring error between prediction and calibration frames, and *obj_loss* for detecting the presence of objects in a grid. In Figure 9, the blue curves represent the loss value of our model, and the orange curves represent the loss value of the original model. Both curves demonstrate the faster convergence and lower losses of our proposed model.

To demonstrate the detective speed of our proposed model, we record the results with respect to the test set. As shown in Table 3, the inference time of our model for an image is 8.1 ms, which is 0.6 lower than that of the original model. And the FPS for YOLOv5_Ghost_CA is more than YOLOv5. Through our improvement, under the premise that the model accuracy is slightly improved, our model size is reduced by nearly half and detection speed is also improved.

**Table 3:** Model testing results.

**Model**	**Inference time (ms)**	**FPS**
YOLOv5	8.7	115
YOLOv5_Ghost_CA	8.1	123

#### Ablation experiments

To verify the rationality and indispensability of each section within the improved model, we split the Ghost and CA into separate experiments to assess the individual parts of the model. We perform the experimental evaluations of the YOLOv5 model by adding only Ghost and only CA, respectively.

Table 4 shows that the influences of Ghost to YOLOv5 by a linear transformation to generate Ghost feature maps is effective, significantly reducing network redundancy and diminishing computational complexity. However, it results in a lower mAP value. To address this shortcoming, we choose CA to improve the model detection accuracy. Compared to the original model, the accuracy improves by 0.3% in map@0.5 after adding CA, making up for the loss incurred by the Ghost module. Therefore, it is desirable to incorporate CA and Ghost together into the YOLOv5 model. The experimental results unexpectedly verify our conjecture.

**Table 4:** Comparison results of ablation experiments.

**Model**	**mAP@0.5**	**mAP@0.5:0.95**	**Weights (MB)**	**GFLOPs**	**Parameters (M)**
YOLOv5	98.4	84.9	15.4	15.8	7.020913
YOLOv5_Ghost	98.3	84.6	7.9	8.1	3.683817
YOLOv5_CA	98.7	84.8	14.6	15.9	7.045521

#### Comparative experiments

To demonstrate the uniqueness of CA, we conduct a series of comparative experiments. The results are illustrated in Table 5. We compare CA with other lightweight attention methods, including the extensively adopted SE and CBAM. Under the same experimental conditions, we add them separately to the YOLOv5 network, which was modified by Ghost previously.

**Table 5:** The results of the comparison test.

**Model**	**mAP@0.5**	**mAP@0.5:0.95**	**Parameters (M)**
YOLOv5_Ghost_SE	98.4	84.2	3.700201
YOLOv5_Ghost_CBAM	98.6	84.3	3.716683
YOLOv5_Ghost_CA	98.8	84.5	3.708425

As shown in Table 5, the network model's interpolation performance is improved to various degrees by adding the attention module. Comparing the influences of the three attention mechanisms, we find that SE brings little accuracy improvement because it only considers the channels. In addition, the accuracy of the proposed model is significantly enhanced, as reflected by 0.4% higher mAP@0.5 and 52.6% higher accuracy density than the original model, demonstrating the improved model's validity.

The benefit of CBAM for this model is a 0.4% increase in mAP@0.5, but it is still not the best choice for improvement. First, it captures only local information. Second, it employs the most model parameters since large convolution kernels exist inside the module. In addition, CA employs two complementary one-dimensional global pools to establish long-term spatial dependencies with more comprehensive global information. Therefore, unlike SE, which negatively impacts the network, CA has a 0.4% improvement in mAP@0.5 and 0.3% in mAP@0.5:0.95. At the same time, the mAP@0.5 of CA is 0.2% higher than CBAM while employing fewer model parameters.

**Figure 10:**
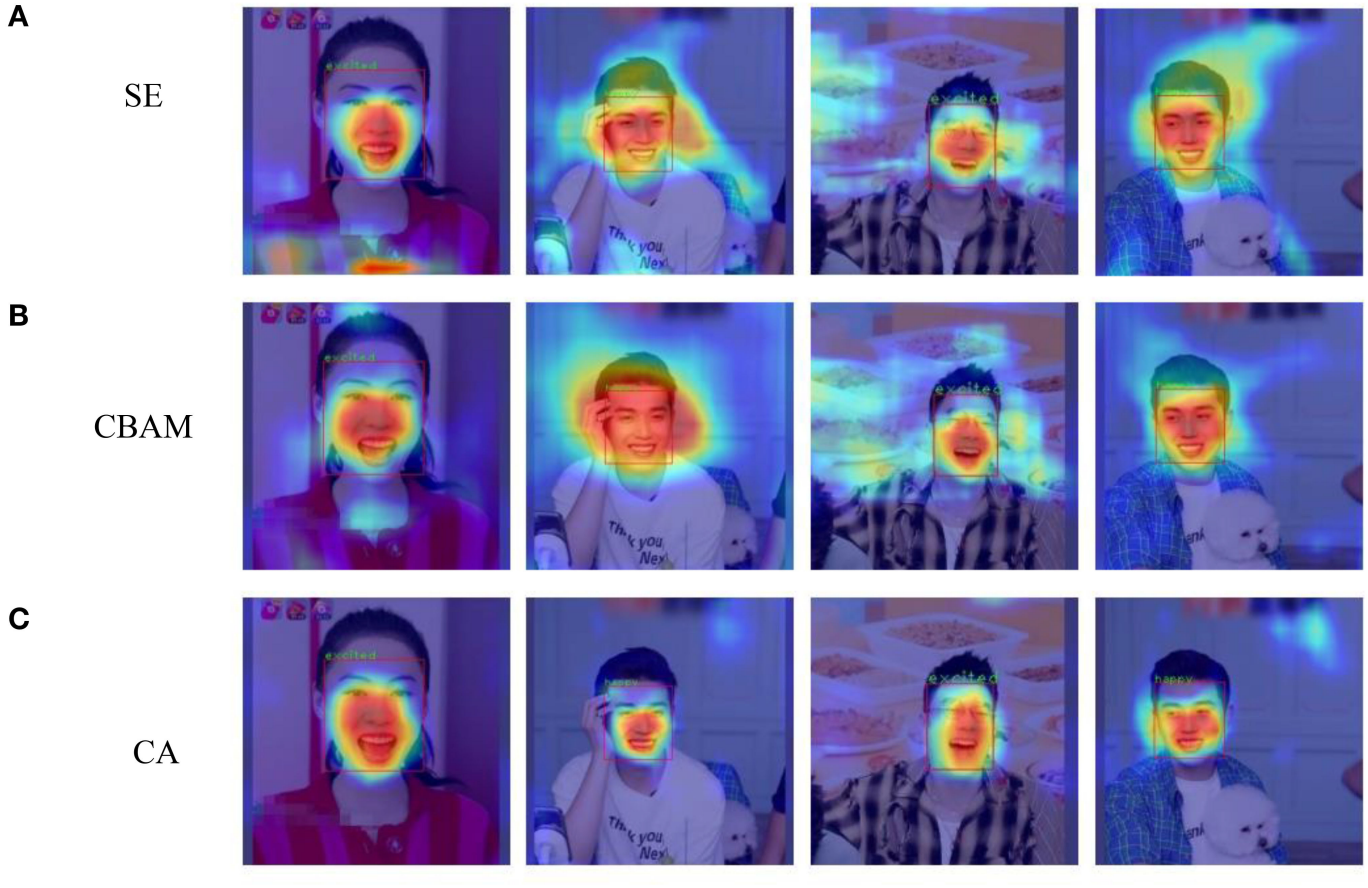
Visualization of learning effects for different attention mechanisms combined with the YOLOv5 model: **(A)** SE; **(B)** CBAM; **(C)** CA.

The feature learning effects of these three attention methods can be compared by visualizing the feature maps of training results using class activation mapping (CAM) (Selvaraju et al., 2017), which not only verifies whether the model overmatches targets but also reveals whether the prediction results are based on image features or backgrounds. From Figure 10, it can be concluded that the allocation of SE is too scattered, so it fails to distinguish well between the facial and background areas. Moreover, although CBAM can focus more on the facial region in the picture, the target range expands greatly. By contrast, CA can precisely focus on the regions of five facial sensory organs, facilitating better learning of facial features.

## Conclusions and future work

In this paper, we have intensively researched efficient feature extraction structure and introduced new methods into the YOLOv5 network for facial expression detection in live streaming video. The training of the improved YOLOv5 comprises two stages. First, a two-step cascade classifier and recycler design is constructed to discriminate and remove invalid images from video, and a live stream facial expression dataset is established. Then, GhostNet and CA are included in the training and inference of YOLOv5 to optimize the network. The experimental results have objectively justified that the improved model is superior for various evaluation criteria, such as complexity, precision, speed, and size.

Future areas for valuable research on accuracy ascension and latency alleviation still exist. Disposition on limited-resource devices such as mobile terminals and embedded kits can help extend the structure to other detection and recognition tasks. Furthermore, people mostly receive multimodal data while viewing live streams, including visual, audio, and bullet screen. Compared to only visual frames, it is worthwhile to use multimodal data to understand facial expressions. We plan to include voice and text as well as facial expressions because these also provide valuable emotional cues for purchasing intention in live stream scenarios.

## Data availability statement

The raw data supporting the conclusions of this article will be made available by the authors, without undue reservation.

## Ethics statement

Written informed consent was obtained from the individual(s) for the publication of any potentially identifiable images or data included in this article.

## Author contributions

All authors listed have made a substantial, direct, and intellectual contribution to the work and approved it for publication.

## Funding

This research was supported by the National Natural Science Foundation of China (No. 71974130) and the National Social Science Fund of China (No. 18BGL093).

## Conflict of interest

The authors declare that the research was conducted in the absence of any commercial or financial relationships that could be construed as a potential conflict of interest.

## Publisher's note

All claims expressed in this article are solely those of the authors and do not necessarily represent those of their affiliated organizations, or those of the publisher, the editors and the reviewers. Any product that may be evaluated in this article, or claim that may be made by its manufacturer, is not guaranteed or endorsed by the publisher.
